# Efficacy of Fludora® Fusion (a mixture of deltamethrin and clothianidin) for indoor residual spraying against pyrethroid-resistant malaria vectors: laboratory and experimental hut evaluation

**DOI:** 10.1186/s13071-020-04341-6

**Published:** 2020-09-11

**Authors:** Augustin Fongnikin, Nadia Houeto, Abel Agbevo, Abibath Odjo, Thomas Syme, Raphael N’Guessan, Corine Ngufor

**Affiliations:** 1 London School of Hygiene and Tropical Medicine (LSHTM), London, UK; 2 Centre de Recherches Entomologiques de Cotonou (CREC), Cotonou, Benin; 3 Panafrican Malaria Vector Research Consortium (PAMVERC), Cotonou, Benin

**Keywords:** Clothianidin, Mixtures, Indoor residual spraying, Fludora® Fusion, Pyrethroid resistance, Malaria vectors, Fludora, Formulations, *Anopheles*, Cové, IRS block substrates, Neonicotinoids, IRS mixtures, Experimental huts, Vector control, Mosquito control

## Abstract

**Background:**

A new generation of IRS insecticides which can provide improved and prolonged control of pyrethroid-resistant malaria vector populations are being developed. Fludora® Fusion is a new IRS insecticide containing a mixture of deltamethrin and clothianidin, a neonicotinoid.

**Methods:**

The efficacy of Fludora® Fusion IRS was evaluated over 11–12 months on concrete and mud substrates in laboratory bioassays and experimental huts against wild free-flying pyrethroid-resistant *Anopheles gambiae* (*sensu lato*) in Cové, Benin. A comparison was made with the two active ingredients of the mixture; clothianidin and deltamethrin, applied alone. CDC bottle bioassays were also performed to investigate resistance to clothianidin in the wild vector population.

**Results:**

Fludora® Fusion induced > 80% laboratory cone bioassay mortality with both susceptible and pyrethroid-resistant *An. gambiae* (*s.l.*) for 7–9 months on concrete block substrates and 12 months on mud block substrates. The vector population at the experimental hut site was fully susceptible to clothianidin in CDC bottle bioassays. Overall mortality rates of wild free-flying pyrethroid-resistant *An. gambiae* (*s.l.*) entering the experimental huts during the 11-month trial were < 15% with deltamethrin and significantly higher with Fludora® Fusion (69–71%) and clothianidin alone (72–78%). Initial high experimental hut mortality rates with Fludora® Fusion (> 80%) only declined by 50% after 8 months. Monthly *in situ* wall cone bioassay mortality of susceptible mosquitoes was > 80% for 9–12 months with Fludora® Fusion and clothianidin alone. Fludora® Fusion induced significantly higher levels of early exiting of mosquitoes compared to clothianidin alone (55–60% vs 37–38%, *P* < 0.05).

**Conclusions:**

Indoor residual spraying with Fludora® Fusion induced high and prolonged mortality of wild pyrethroid-resistant malaria vectors for 7–10 months mostly due to the clothianidin component and substantial early exiting of mosquitoes from treated huts due to the pyrethroid component. Fludora® Fusion is an important addition to the current portfolio of IRS insecticides with the potential to significantly reduce transmission of malaria by pyrethroid-resistant mosquito vectors. 
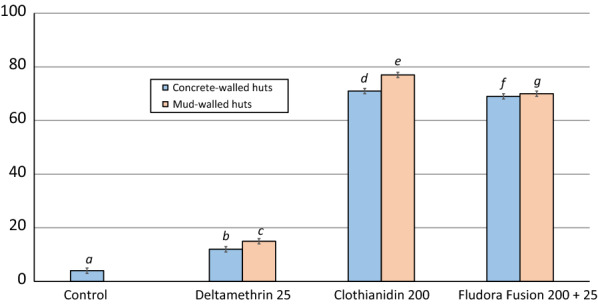

## Background

Indoor residual spraying remains a core strategy in the fight against malaria due to its ability to rapidly reduce transmission [1]. It constitutes the application of a residual insecticide to potential resting surfaces of malaria vectors; usually the inner walls, ceiling and eaves of human habitats and domestic animal shelters. There has been a substantial increase in the use of IRS over the last two decades [2, 3] and this has contributed significantly to reductions in malaria morbidity and mortality observed in many endemic countries [4]. The effectiveness of IRS for malaria vector control however depends on several factors; mainly the continued susceptibility of local vectors to the insecticides deployed and the duration of its action on treated home wall substrates. For some decades, IRS relied heavily on a rather limited number of classes of insecticides [2, 3] most of which were short-lived on home wall substrates (2–5 months) thus requiring multiple resource-demanding IRS campaign rounds when used in areas with stable malaria transmission [5]. Malaria vectors have also developed resistance to these conventional insecticides which is now widespread and increasing in intensity across Africa [6] and this, together with their short residual effect is driving the development of a new generation of long-lasting IRS insecticides to which local vectors are largely susceptible.

The neonicotinoid, clothianidin is a new repurposed insecticide which was recently added to the WHO’s list of pre-qualified insecticides for use in indoor residual spraying [7]. Clothianidin presents a new mode action which differs from that of conventional public health insecticides acting as an agonist on nicotinic acetylcholine receptors (nAChR) [8]. Owing to its novel mode of action, it shows potential to provide improved control of vector populations that have developed resistance to older public health insecticides. The addition of clothianidin to the portfolio of IRS insecticides also provides an opportunity to mitigate the development and spread of insecticide resistance in malaria vectors through the rotational use of IRS insecticides and the development of mixture IRS co-formulations [9]. Insecticide mixtures for IRS need to be explored with new public health insecticides when they become available because mixtures have the dual potential to improve malaria vector control through the combined effect of both active ingredients and contribute to insecticide resistance management, especially in areas where resistance to both active ingredients is not yet established [10].

In a previous experimental hut study in Benin, a tank mix of clothianidin and deltamethrin induced high and prolonged mortality (8–9 months) in wild pyrethroid-resistant *An. gambiae* (*s.l.*) owing to the clothianidin component and early exiting of mosquitoes from experimental huts due to the pyrethroid component [11]. The encouraging results from this early proof of concept study led to the development of Fludora® Fusion (Bayer CropScience, Monheim, Germany), a new IRS formulation of a wettable powder product containing 500 g/kg of clothianidin and 62.5 g/kg of deltamethrin in water-soluble sachets. In the present study, the efficacy of Fludora® Fusion was evaluated against pyrethroid-resistant *An. gambiae* (*s.l.*) in southern Benin under both laboratory and experimental hut conditions. Its residual effect was assessed on mud and concrete wall substrates. A comparison was made with the two active ingredients of the mixture; clothianidin and deltamethrin, applied alone.

## Methods

### Laboratory evaluation

#### Preparation and treatment of block substrates

Concrete and mud blocks used for the laboratory bioassays were formed in 9 cm Petri dishes and dried at 27 ± 2 °C and 80 ± 10% RH for 30 days before insecticide application. Concrete blocks were made by mixing cement with sand at a 1:1 ratio while mud blocks were made from local mud paste to which 10% cement was added to improve its hardness, in line with local practices. These substrates were treated using a potter tower sprayer to achieve a homogeneous and accurate deposit of the target concentration of active ingredient per unit area. Blocks were weighed before and after treatment to ensure the target insecticide volume was delivered. All treated blocks were stored, unsealed at 30 ± 2 ℃, 80 ± 10% RH in between bioassays. Four replicate blocks of each substrate were treated with each of the following insecticides: Fludora® Fusion WP at 200 mg + 25 mg/m^2^; Clothianidin WG at 200 mg/m^2^; and Deltamethrin WG at 25 mg/m^2^.

### Residual efficacy on treated block substrates

Residual efficacy of the insecticides on the treated mud and concrete block substrates were assessed in monthly cone bioassays following WHO guidelines [12]. Forty (40) 2–5-day-old laboratory maintained susceptible *Anopheles gambiae* Kisumu and pyrethroid-resistant *Anopheles gambiae* (*s.l.*) Cové mosquitoes were exposed for 30 min to each treatment and substrate in replicates of 10 mosquitoes per block. The laboratory cone bioassays were conducted at monthly intervals for up to 12 months. Based on the delayed effect of clothianidin on mosquito mortality as demonstrated in previous studies [11], mortality was recorded every 24 hours up to 120 hours post-exposure across all experiments.

### Experimental hut trial

#### Study site and experimental huts

The hut trial was performed at the CREC/LSHTM experimental hut station in Cové, Southern Benin. The field site is located in an irrigated valley producing rice almost year-round and providing suitable breeding habitats for mosquitoes. The rainy season extends from March to October and the dry season from November to February. The vector population consists of both *An. coluzzii* and *An. gambiae* (*sensu stricto*) with the latter occurring at lower frequencies (~23%) and mostly in the dry season [13]. The vector population is highly resistant to pyrethroids. Molecular genotyping and microarray studies have demonstrated a high frequency of the L1014F allele (> 90%) and overexpression of the cytochrome P450s CYP6P3, associated with pyrethroid detoxification [13]. The trial ran for 11 months between December 2015 and November 2016 in 7 experimental huts of West African design. The experimental huts are made from concrete bricks with a corrugated iron roof. Inner walls were plastered with either concrete or mud and the ceilings fitted with palm thatch matting. Each hut was built on a concrete plinth surrounded by a water-filled moat to prevent the entry of scavenging ants and had a wooden framed veranda trap to capture exiting mosquitoes. Mosquito entry occurred *via* four window slits each measuring 1 cm and situated on three sides of the hut.

#### Susceptibility of wild vector population to clothianidin

CDC bottle bioassays were performed to investigate the susceptibility of the Cové vector population to clothianidin. Wild pyrethroid-resistant *An. gambiae* Cové adult mosquitoes which emerged from larvae collected from breeding sites within the experimental hut site were exposed to 250 ml Wheaton bottles treated with clothianidin at 90 µg/bottle and deltamethrin at 12.5 µg/bottle. Stock solutions were prepared using a mixture of acetone and 81% rapeseed oil (methyl ester) in the ratio of 2:3 as a solvent for clothianidin and acetone alone for deltamethrin. Four replicate bottles were prepared per insecticide and approximately 100 2–5-days-old F1 wild pyrethroid-resistant *An. gambiae* (*s.l.*) Cové were tested against each insecticide in replicates of 25 mosquitoes per bottle. The CDC bottle bioassay protocol was modified; mosquitoes were exposed for 2 h after which they were transferred to observation cups and knockdown recorded 1 h after exposure and mortality after every 24 h for up to 120 h. Tests were also performed with the susceptible *An. gambiae* Kisumu strain for comparison.

#### Experimental hut treatments

The following insecticides were tested in the experimental huts: (i) Fludora® Fusion Wettable Powder containing 500 g/kg clothianidin + 62.5 g/kg deltamethrin (Bayer CropScience, Germany); (ii) K-Othrine Water Dispersible Granule containing 250 g/kg deltamethrin (Bayer CropScience, Germany); (iii) Clothianidin Water Dispersible Granule containing 700 g/kg (Bayer CropScience, Germany).

The following treatments and application rates were compared in 7 experimental huts: (i) Unsprayed hut (control)–concrete-walled hut; (ii) Deltamethrin applied at 25 mg/m^2^–concrete-walled hut; (iii) Deltamethrin applied at 25 mg/m^2^–mud-walled hut; (iv) Clothianidin applied at 200 mg/m^2^–concrete-walled hut; (v) Clothianidin applied at 200 mg/m^2^–mud-walled hut; (vi) Fludora® Fusion applied at 200 mg + 25 mg/m^2^–concrete-walled hut; and (vii) Fludora® Fusion applied at 200 mg + 25 mg/m^2^– mud-walled hut.

The walls and ceiling of each experimental hut were sprayed using a Hudson X-pert compression sprayer. To improve spraying accuracy, spray swaths were pre-marked on hut walls and a guidance pole was attached to the end of the spray lance to maintain a fixed distance to the wall.

#### Assessing spray quality

Before spraying, filter papers (Whatman No 1) measuring 5 × 5 cm were fixed on each hut wall using masking tape. After spraying, the filter papers were removed, carefully packaged in Aluminium foil and stored at 4 ℃ for about 2 weeks after which they were shipped to BioGenius GmBH, Germany, for chemical analysis to assess the quality of the spray applications using gas chromatography.

#### Hut trial procedure

The trial followed the WHO guidelines for evaluation of IRS products [12]. Treatments were randomly allocated to experimental huts. Seven consenting adult human volunteers slept in the huts from 21:00 to 5:00 h each trial night to attract mosquitoes and were rotated between huts on successive nights to adjust for variation in individual attractiveness to mosquitoes. In the morning, mosquitoes were collected from the room and veranda using aspirators and brought to the laboratory where they were identified and scored as fed or unfed and dead or alive. Live mosquitoes were provided with 10% glucose solution and mortality scored every 24 h for up to 120 h.

#### Outcome measures

The efficacy of each experimental hut treatment was expressed in terms of the following outcome measures: (i) Exiting rates (the proportion of mosquitoes collected in the veranda); (ii) Blood-feeding rates (the proportion of blood-fed mosquitoes); and (iii) Mortality (the proportion of mosquitoes found dead after a 120 h holding time).

#### Residual activity of insecticide treatments

To assess the residual activity of the treatments on the treated experimental hut walls, WHO cone bioassays were conducted using 2–5-day-old, female mosquitoes of the insecticide susceptible *An. gambiae* Kisumu strain. Bioassays were performed 3 days after application of treatments and at monthly intervals thereafter over 12 months. A total of 50 mosquitoes were tested per hut in cohorts of 10 per cone on each treated wall/ceiling surface. Mosquitoes were exposed to treated surfaces for 30 min following WHO guidelines [12]. Mortality was recorded every 24 h up to 120 h post-exposure.

### Data analysis

Experimental hut data were entered in Excel and transferred to Stata 15.1 for analysis. Proportional data (exiting rate, blood-feeding and mortality) were analysed using logistic regression while adjusting for the effects of sleeper attractiveness to mosquitoes. Cone bioassay mortality was pooled for each treatment and substrate at each time point and compared against an 80% cut-off criteria following WHO guidelines [12].

## Results

### Laboratory cone bioassay results

**Fig. 1:**
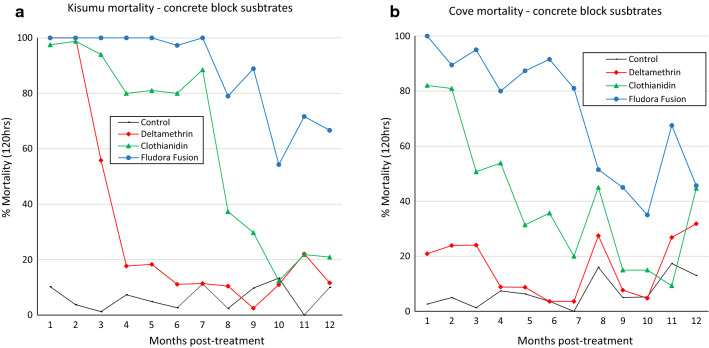
Monthly mortality of susceptible *Anopheles gambiae* Kisumu (**a**) and pyrethroid-resistant *Anopheles gambiae* (*s.l.*) Cové (**b**) exposed to insecticide-treated concrete block substrates in WHO cone bioassays. Each data point represents monthly mortality 120 h post-exposure

**Fig. 2:**
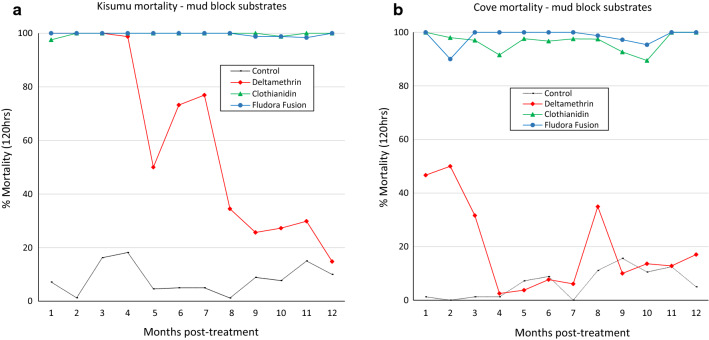
Monthly mortality of susceptible *Anopheles gambiae* Kisumu (**a**) and pyrethroid-resistant *Anopheles gambiae* (*s.l.*) Cové (**b**) exposed to insecticide-treated mud block substrates in WHO cone bioassays. Each data point represents monthly mortality 120 h post-exposure

Mortality of susceptible *An. gambiae* Kisumu and pyrethroid-resistant *An. gambiae* (*s.l.*) Cové strains in laboratory cone bioassays with control untreated mud and concrete blocks did not exceed 20% at any time point (Figs. 1 and 2). For both types of substrates, mortality rates for each treatment were generally higher/more persistent with the Kisumu strain compared to the pyrethroid-resistant Cové strain. Mortality with deltamethrin-treated blocks was > 80% for the first 2–4 months with the susceptible Kisumu strain after which it declined sharply (Figs. 1a and 2a), but with pyrethroid-resistant Cové strain, mortality on both substrate-types did not exceed 50% at any time point (Figs. 1b and 2b). On concrete blocks, mortality with clothianidin was > 80% for 7 months with the susceptible Kisumu strain (Fig. 1a) and only 2 months with the pyrethroid-resistant Cové strain (Fig. 1b). Meanwhile, Fludora® Fusion induced more residual mortality on concrete blocks which remained > 80% for 9 months with the susceptible Kisumu strain (Fig. 1a) and 7 months with the pyrethroid-resistant Cové strain (Fig. 1b). With mud blocks, mortality rates with clothianidin and Fludora® Fusion were very stable remaining above 90% for both mosquito strains for up to 12 months (Fig. 2).

### Experimental hut results

#### Susceptibility of vector population to clothianidin

**Fig. 3:**
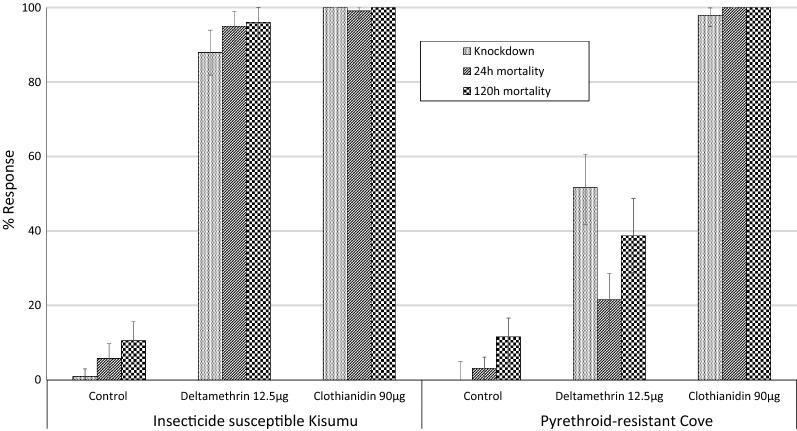
Susceptibility of wild pyrethroid-resistant *An. gambiae* (*s.l.*) from Cové to clothianidin in CDC bottle bioassays. ~ 100 2–5-days-old mosquitoes of each strain were exposed to each insecticide in 4 replicate bottles. Error bars represent 95% confidence intervals

Results from the CDC bottle bioassays are presented in Fig. 3. Mortality rates with the control were less than 10% even up to 120 h post-exposure. Knockdown and mortality of wild F1 *An. gambiae* (*s.l.*) mosquitoes emerging from larvae collected from the Cové experimental hut site were respectively 52% and 39% with deltamethrin-treated bottles thus confirming the levels of pyrethroid resistance in the Cové vector population. With clothianidin-treated bottles, knockdown and mortality after 24 h were respectively 98% and 100%, demonstrating full susceptibility to the insecticide. Meanwhile, with the susceptible *An. gambiae* Kisumu strain, mortality was > 95% at 24 h with both insecticides. More detailed results on CDC bottle bioassays are available in the supplementary information (Additional file 1: Table S1).

#### Mosquito entry and exiting

The experimental hut results are summarised in Table 1. A total of 57,518 wild free-flying pyrethroid-resistant female *An. gambiae* (*s.l.*) mosquitoes were collected in the experimental huts over the 11-month trial indicating an average of 8217 mosquitoes per hut (Table 1). For both concrete and mud-walled huts, exiting rates with deltamethrin (62–64%) and Fludora® Fusion (55–60%) was significantly higher than the control hut (40%) and clothianidin-only huts (37–38%) (*P* < 0.05). The exiting rates observed with Fludora® Fusion-treated huts are therefore attributable to the deltamethrin component in the mixture. Blood-feeding rates were high across all insecticide treatments tested (87–92%); hence, there was no evidence of blood-feeding inhibition with any of these treatments regardless of the wall substrate (Table 1).

**Table 1:** Experimental hut results for wild free-flying pyrethroid-resistant *An. gambiae* (*s.l*.) entering IRS-treated experimental huts in Cové, Benin

Wall type	Hut treatment	Total collected	%Exiting (95% CI)	%Blood fed (95% CI)	%Mortality (120 h) (95% CI)
Concrete walls	Control	10,917	40 (39–41)^a^	87 (86–88)^a^	4 (3–4)^a^
	Deltamethrin (25 mg/m^2^)	8674	62 (61–63)^b^	91 (90–92)^b^	12 (11–13)^b^
	Clothianidin (200 mg/m^2^)	8040	38 (37–39)^c^	90 (89–91)^bc^	72 (71–73)^c^
	Fludora® Fusion (200 mg/m^2^)	8149	60 (59–61)^d^	91 (90–91)^bd^	69 (68–70)^d^
Mud walls	Deltamethrin (25 mg/m^2^)	6632	64 (62–65)^b^	92 (91–93)^e^	15 (15–16)^e^
	Clothianidin (200 mg/m^2^)	6290	37 (36–38)^c^	89 (88–90)^cd^	78 (77–79)^f^
	Fludora® Fusion (200 mg/m^2^)	8562	55 (54–56)^e^	87 (86–88)^a^	70 (69–71)^g^

*Notes*: Values bearing the same superscript letter along a column are not significantly different at the 5% level (*P* > 0.05, logistic regression)

#### Mortality rates

**Fig. 4:**
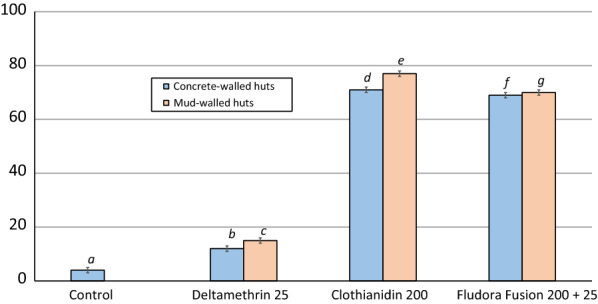
Overall mortality (%) of wild pyrethroid-resistant *An. gambiae* (*s.l.*) entering IRS-treated experimental huts in Cové, Benin. Each bar represents mortality over 11 months. Bars bearing the same letter label are not significantly different at the 5% level (*P* > 0.05, logistic regression). Error bars represent 95% confidence intervals. *Abbreviations*: Delta, deltamethrin; Cloth, clothianidin

**Fig. 5:**
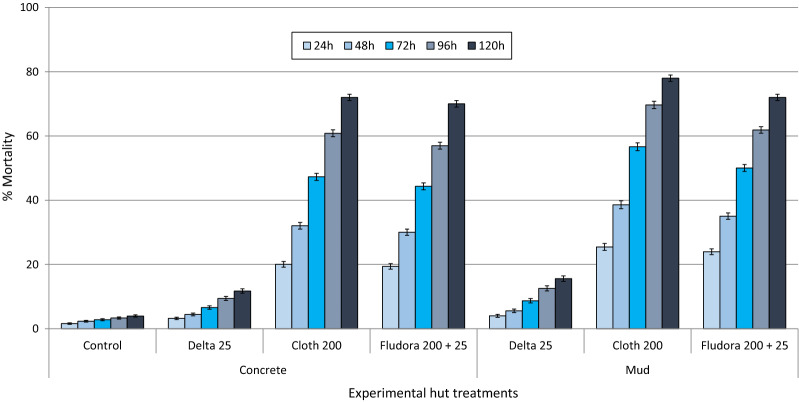
Delayed mortality (%) of wild pyrethroid-resistant *An. gambiae* (*s.l.*) entering IRS-treated experimental huts in Cové, Benin. Each bar represents % overall mortality every 24 hours up to 120 hours for each treatment over 11 months. Error bars represent 95% confidence intervals. *Abbreviations*: *Delta* deltamethrin; *Cloth* clothianidin

Mortality rates of wild free-flying pyrethroid-resistant *An. gambiae* (*s.l.*) which entered the experimental huts during the 11-month trial are presented in Table 1 and Figs. 4, 5.

*Overall mortality rates*. Overall mortality was 4% in the control hut and 12–15% in the deltamethrin IRS-treated huts. Overall mortality in Fludora® Fusion (69–70%) and clothianidin-sprayed huts (72–78%) was significantly higher than in deltamethrin-treated huts (12–15%; *P* < 0.001). Clothianidin-sprayed huts however killed significantly larger proportions of mosquitoes compared to Fludora® Fusion (*P* < 0.05). As observed in laboratory bioassays, mortality rates with all insecticide treatments were generally higher in mud-walled huts compared to concrete-walled huts (*P* < 0.01), nevertheless, the actual differences were generally small (1–6 units).

*Delayed mortality*. Figure 5 presents the mortality rates of wild pyrethroid-resistant *An. gambiae* (*s.l.*) in the experimental huts recorded every 24 h up to 120 h. Mortality with the control was < 2% after 24 h and increased to only 4% after 120 h. For both types of wall substrates, Fludora® Fusion and clothianidin alone demonstrated a substantial increase in delayed mortality with the numbers of days after collection from the treated huts. Mortality in huts treated with clothianidin and Fludora® Fusion increased steadily from approximately 20–25% after 24 h to 69–78% after 120 h. This trend was not strongly expressed in huts treated with deltamethrin alone where mortality did not exceed 20% after 120 h for both substrate types, thus demonstrating that the delayed mortality effect observed with Fludora® Fusion is largely due to the clothianidin component of the mixture.

#### Residual efficacy of IRS treatments

**Fig. 6:**
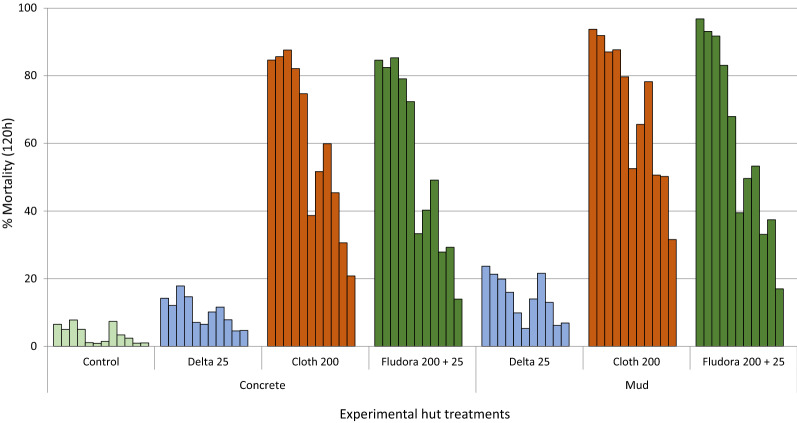
Monthly mortality rates of wild free-flying pyrethroid-resistant *An. gambiae* entering IRS-treated concrete and mud-walled experimental huts in Cové, Benin. Each bar represents % mortality (120 h) over each successive month of the trial for treatments applied in concrete and mud-plastered huts. *Abbreviations*: *Delta* deltamethrin; *Cloth* clothianidin

*Monthly mortality of wild mosquitoes.* Figure 6 shows monthly mortality rates of wild free-flying, pyrethroid-resistant *An. gambiae* (*s.l.*) entering IRS-treated experimental huts over 11 months for both concrete and mud-walled huts. For both substrates, Fludora® Fusion and clothianidin killed at least 80% of wild, pyrethroid-resistant *An. gambiae* (*s.l.*) entering experimental huts for the first 4 months, after which mortality declined progressively to < 40% after 9 months. Conversely, the proportion of wild pyrethroid-resistant *An. gambiae* (*s.l.*) killed in the deltamethrin-treated huts did not exceed 25% throughout the trial.

**Fig. 7:**
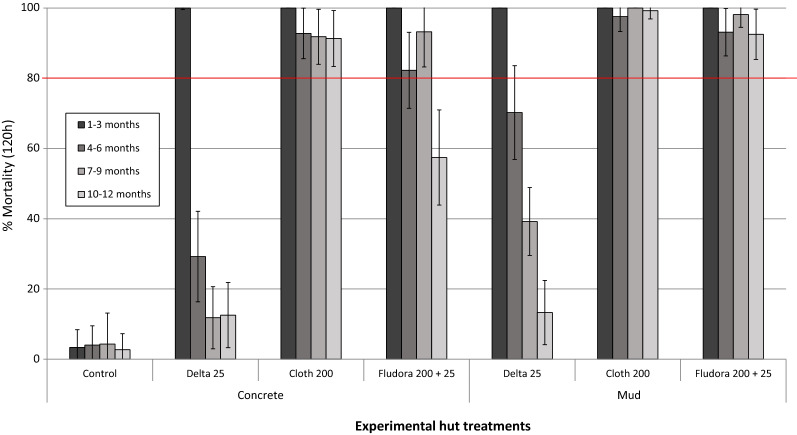
Quarterly cone bioassay mortality of susceptible *An. gambiae* Kisumu on IRS-treated concrete and mud-walled experimental huts in Cové, Benin. Each bar represents pooled mean mosquito mortality over 3 consecutive months. ~ 50 mosquitoes were tested each month in wall cone bioassays in each hut. *Abbreviations*: *Delta* deltamethrin, *Cloth* clothianidin

*Cone bioassay mortality on treated hut walls* Quarterly mortality rates (pooled for every 3 months) of the susceptible, laboratory-maintained *An. gambiae* Kisumu strain following exposure to mud and concrete IRS-treated experimental hut walls in *in situ* cone bioassays are presented in Fig. 7. Cone bioassay mortality on control untreated hut walls did not exceed 5% for all four quarters. With deltamethrin-treated huts, cone bioassay mortality was > 80% only for the first 3 months after which it declined sharply. Cone bioassay mortality remained > 80% for 9 months with Fludora® Fusion and 12 months with clothianidin on concrete-walled huts and 12 months with both insecticides on mud-walled huts.

#### Quality of IRS applications

The results from chemical analysis of filter papers performed at BioGenius GmBH, Germany, are presented in Table 2. The average AI content in filter papers for each treatment and wall substrate type were within an acceptable deviation of 22% from the target dose showing that the treatments were correctly applied.

**Table 2:** Chemical analysis of filter papers from IRS-treated experimental huts in Cové, Benin (mean insecticide content)

Hut treatment	Wall type	Clothianidin content (mg/m^2^)	RSD (%)	%Deviation	Alpha content (mg/m^2^)	RSD (%)	%Deviation
Deltamethrin	Concrete	–	–	–	21	54	16
	Mud	–	–	–	30	15	−20
Clothianidin	Concrete	208	67	− 4	–	–	–
	Mud	244	20	− 22	–	–	–
Fludora® Fusion	Concrete	211	23	− 5.5	26	24	−4
	Mud	186	23	7	23	8	8

*Abbreviation*: RSD, relative standard deviation

## Discussion

This study evaluated the efficacy of Fludora® Fusion, a clothianidin and deltamethrin mixture, for indoor residual spraying in laboratory studies and in an experimental hut trial against a vector population in Benin which is highly resistant to pyrethroids. The low mortality response with deltamethrin IRS in the experimental huts is very typical of studies conducted in this area of Benin [11, 14] thus demonstrating the redundancy of solo pyrethroid products for IRS and further highlighting the need for novel non-pyrethroid IRS insecticides. Studies performed with Fludora® Fusion in West Africa have so far reported its efficacy on treated surfaces only in cone bioassays [15, 16] which generally do not take into consideration the behaviour of vector mosquitoes. Our study demonstrates for the first time the efficacy of Fludora® Fusion against wild free-flying pyrethroid-resistant malaria vectors in household settings in Benin. At all levels of evaluation, Fludora® Fusion clearly showed greatly improved and prolonged overall mortality of pyrethroid susceptible and resistant strains of *An. gambiae* (*s.l.*) compared to deltamethrin on both mud and concrete substrates. The results confirm previous findings across Africa [11, 15, 16], thus demonstrating the suitability of Fludora® Fusion for indoor residual spraying in Benin and other malaria-endemic areas which are characterised by high intensities of pyrethroid resistance in local mosquito vectors.

One rationale for the use of a mixture of insecticides in the Fludora® Fusion IRS formulation over clothianidin alone is the possibility to achieve greater levels of vector control through the combined effects of both active ingredients. In laboratory cone bioassays with concrete block substrates, Fludora® Fusion showed longer residual activity compared to clothianidin alone. This was surprisingly more evident with the pyrethroid-resistant strain where mortality was > 80% for five months with Fludora® Fusion-treated concrete blocks compared to only one month with clothianidin. This suggests a synergistic action of both insecticides with the mixture in cone bioassays which appeared not to be affected by the pyrethroid-resistant status of the mosquito strain. This effect was however not observed in experimental huts; mortality rates of wild pyrethroid-resistant mosquitoes were higher in huts treated with clothianidin alone compared to Fludora® Fusion; though this was only by a few units. The difference in outcome could be attributed to differences in mosquito behaviour in reaction to the insecticides in cone bioassays compared to experimental huts. Contrary to cone bioassays where mosquito movement is very limited, mosquitoes enter experimental huts *ad libitum* and feed on the sleeper before resting on the IRS-treated walls to pick up the IRS treatment. Therefore, the high excito-repellent effect of deltamethrin in Fludora® Fusion could have prevented mosquitoes from resting long enough on the treated hut walls to pick up adequate amounts of clothianidin in the mixture IRS thus leading to reduced overall mortality rates compared to clothianidin.

Previous experimental hut studies also showed significantly reduced mortality with an IRS mixture of chlorfenapyr and alpha-cypermethrin compared to chlorfenapyr alone (43% vs 63% [14] and 18–22% vs 38–46% [17]) which was also attributed to the irritant effect of the pyrethroid in the mixture. Alternatively, the small difference in performance between Fludora® Fusion and clothianidin-solo IRS in huts could also be due to differences in the types of IRS formulations (WG for clothianidin and WP for Fludora® Fusion) as has been previously reported with some pyrethroid IRS insecticides [18, 19]. However, contrary to the chlorfenapyr and alpha-cypermethrin mixture, the mortality achieved with Fludora® Fusion was only a few points lower than that of clothianidin alone (70–71% vs 72–78%) and this is less expected to result in operationally significant differences in the impact on clinical malaria when used in IRS campaigns. Also, the high insecticide induced exiting rate observed with Fludora® Fusion compared to clothianidin alone in this study and previously [11] is important for reducing indoor resting and biting which may contribute to lowering transmission intensities.

While a universal diagnostic dose for clothianidin is yet to be established for malaria vectors, our results showed full susceptibility to clothianidin in CDC bottle bioassays at 90 µg/bottle in a vector population that is highly resistant pyrethroids [13]. Recent studies in neighbouring countries in West Africa also reported full susceptibility to clothianidin [20, 21] at even lower doses [21] in pyrethroid-resistant malaria vectors despite high levels of resistance to other neonicotinoids widely used in agriculture in the region [21]. As new active ingredients are introduced into the public health portfolio of insecticides, strategies to delay the development of resistance to these insecticides in malaria vectors must be taken into consideration before large-scale use in order to extend their useful life [9]. Modelling studies have suggested that the use of an insecticide in a mixture especially when it is effective at killing mosquitoes, has potential to prompt slower evolution of resistance to the insecticide compared to when it is used alone [22]. Considering its effectiveness, the co-formulation of clothianidin into the Fludora® Fusion mixture might be a better option for delaying the development and spread of resistance to clothianidin in malaria vectors compared to formulations with clothianidin alone. Further studies to investigate this hypothesis under large scale use would be necessary.

Contrary to a previous study in Benin [15], we observed a longer residual effect of Fludora® Fusion on mud walls compared to concrete surfaces. This could be attributed to the addition of a small amount of cement in the mud paste used for moulding the blocks and wall plaster used in our study. Previous studies examining the content of mud from Cové, Benin, demonstrated a high silt content which makes it less suitable for construction (unpublished data). The addition of a small amount of cement is in line with a common local practice in Benin and other parts of Africa which is aimed at improving the durability and aesthetic of inner wall plastering in earthen houses [23]. This could have changed the characteristics of the mud substrates making them more stable, much less porous and more suitable for Fludora® Fusion compared to the traditional mud-plastered wall. This finding shows that Fludora® Fusion could be highly effective in semi-urban and usually more populated areas in many African settings where the addition of cement to mud for construction and plastering of earthen houses is becoming common [23].

Based on results from our studies and others, Fludora® Fusion was added to the WHO’s list of pre-qualified IRS formulations for vector control becoming the first IRS insecticide to contain a mixture of active ingredients [7]. Fludora® Fusion was recently deployed for IRS in Benin by the National Malaria Control Programme in the 2020 IRS campaign; studies to assess its impact under operational conditions are on-going.

## Conclusions

Fludora® Fusion when applied for IRS induced high mortality against wild free-flying pyrethroid-resistant malaria vectors in Benin mostly due to the clothianidin component and early exiting due to the deltamethrin component. The insecticide also provided prolonged indoor mosquito vector control which lasted for 7–10 months on both mud and concrete substrates. Fludora® Fusion proves to be an important addition to the current portfolio of IRS insecticides.

## Supplementary information

**Additional file 1: Table S1.** Knockdown and mortality of pyrethroid-resistant *An. gambiae* (*s.l.*) Cové, Benin, in CDC bottle bioassays with clothianidin.

## Data Availability

The datasets used and/or analysed during the present study are available from the corresponding author upon reasonable request.

## Abbreviations

IRS: indoor residual spraying
WHO: World Health Organization
PQ: prequalification team
GPIRM: Global Plan for Insecticide Resistance Management
WHOPES: WHO Pesticide Evaluation Scheme
CREC: Centre de Recherche Entomologique de Cotonou
LSHTM: London School of Hygiene & Tropical Medicine
Kdr: knockdown resistance
