# Supplementary material for: Efficacy of Fludora® Fusion (a mixture of deltamethrin and clothianidin) for indoor residual spraying against pyrethroid-resistant malaria vectors: laboratory and experimental hut evaluation
Source: Parasit Vectors. 2020 Sep 11;13:466. doi: 10.1186/s13071-020-04341-6 (PMC7488472; doi:10.1186/s13071-020-04341-6)
Supplement: Supplementary file 1 — Additional file 1: Table S1. Knockdown and mortality of pyrethroid-resistant An. gambiae (s.l.) Cové, Benin, in CDC bottle bioassays with clothianidin. [file 13071_2020_4341_MOESM1_ESM.docx]

**Additional file 1**

**Efficacy of Fludora® Fusion (a mixture of deltamethrin and clothianidin) for indoor residual spraying against pyrethroid-resistant malaria vectors; laboratory and experimental hut evaluation.**

Augustin Fongnikin^2, 3^, Nadia Houeto^2, 3^, Abel Agbevo^2, 3^, Abibath Odjo^2, 3^, Thomas Syme^1^, Raphael N’Guessan^1^, Corine Ngufor^1, 2, 3*^

**Table S1:** Knockdown and mortality of pyrethroid-resistant *An gambiae* sl Cove, Benin in CDC bottle bioassays with clothianidin

|  |  |  | % Knockdown | % Mortality | | | | |
| --- | --- | --- | --- | --- | --- | --- | --- | --- |
| Mosquito strain | Insecticide | N exposed | 1 hour | 24h | 48h | 72h | 96h | 120h |
| Susceptible *An gambiae* Kisumu | Control | 104 | 1 | 6 | 9 | 9 | 11 | 11 |
|  | Deltamethrin 12.5µg | 99 | 88 | 95 | 96 | 96 | 96 | 96 |
|  | Clothianidin 90µg | 103 | 100 | 99 | 100 | 100 | 100 | 100 |
| Pyrethroid-resistant *An gambiae* Cove | Control | 95 | 0 | 3 | 5 | 6 | 8 | 12 |
|  | Deltamethrin 12.5µg | 93 | 52 | 22 | 26 | 27 | 28 | 39 |
|  | Clothianidin 90µg | 93 | 98 | 100 | 100 | 100 | 100 | 100 |
